# Experimental and Theoretical Study of Radiation Shielding Features of CaO-K_2_O-Na_2_O-P_2_O_5_ Glass Systems

**DOI:** 10.3390/ma14143772

**Published:** 2021-07-06

**Authors:** M. I. Sayyed, Badriah Albarzan, Aljawhara H. Almuqrin, Ahmed M. El-Khatib, Ashok Kumar, Daria I. Tishkevich, Alex V. Trukhanov, Mohamed Elsafi

**Affiliations:** 1 Department of Physics, Faculty of Science, Isra University, Amman 11622, Jordan; 2 Department of Nuclear Medicine Research, Institute for Research and Medical Consultations (IRMC), Imam Abdulrahman bin Faisal University (IAU), Dammam 31441, Saudi Arabia; 3 Department of Physics, College of Science, Princess Nourah Bint Abdulrahman University, Riyadh 11671, Saudi Arabia; Baalbarzan@pnu.edu.sa (B.A.); ahalmoqren@pnu.edu.sa (A.H.A.); 4 Physics Department, Faculty of Science, Alexandria University, Alexandria 21511, Egypt; elkhatib60@yahoo.com (A.M.E.-K.); mohamedelsafi68@gmail.com (M.E.); 5 Department of Physics, University College, Benra, Dhuri 148024, Punjab, India; ajindal9999@gmail.com; 6 Department of Physics, Punjabi University, Patiala 147002, Punjab, India; 7 Laboratory of Magnetic Films Physics, Scientific and Practical Materials Research Centre of National Academy of Sciences of Belarus, 220072 Minsk, Belarus; dashachushkova@gmail.com (D.I.T.); truhanov86@mail.ru (A.V.T.); 8 Laboratory of Single Crystal Growth, South Ural State University, 454080 Chelyabinsk, Russia

**Keywords:** radiation shielding glasses, radiation protection efficiency, mean free path, XCOM software

## Abstract

The gamma radiation shielding ability for CaO-K_2_O-Na_2_O-P_2_O_5_ glasses were experimentally determined between 0.0595 and 1.41 MeV. The experimental MAC results were compared with theoretical results obtained from the XCOM software to test the accuracy of the experimental values. Additionally, the effect of increasing the P_2_O_5_ in the glass composition, or reducing the Na_2_O content, was evaluated at varying energies. For the fabricated glasses, the experimental data strongly agreed with the XCOM results. The effective atomic number (Z_eff_) of the fabricated glasses was also determined. The Z_eff_ values start out at their maximum (12.41–12.55) at the lowest tested energy, 0.0595 MeV, and decrease to 10.69–10.80 at 0.245 MeV. As energy further increases, the Z_eff_ values remain almost constant between 0.344 and 1.41 MeV. The mean free path (MFP) of the fabricated glasses is investigated and we found that the lowest MFP value occurs at the lowest tested energy, 0.0595 MeV, and lies within the range of 1.382–1.486 cm, while the greatest MFP can be found at the highest tested energy, 1.41 MeV, within the range of 8.121–8.656 cm. At all energies, the KCNP40 sample has the lowest MFP, while the KCNP60 sample has the greatest. The half value layer (HVL) for the KCNP-X glasses is determined. For all the selected energies, the HVL values follow the order of KCNP40 < KCNP45 < KCNP50 < KCNP55 < KCNP60. The HVL of the KCNP50 sample increased from 0.996 to 2.663, 3.392, 4.351, and 5.169 cm for energies of 0.0595, 0.245, 0.444, 0.779, and 1.11 MeV, respectively. The radiation protection efficiency (RPE) results reveal that decreasing the P_2_O_5_ content in the glasses improves the radiation shielding ability of the samples. Thus, the KCNP40 sample has the best potential for photon attenuation applications.

## 1. Introduction

Glasses are increasingly being used as protective materials in applications that utilize radiation to absorb incoming photons that may harm workers and patients surrounding the radioactive source. Radiation is currently being used in hundreds of applications spanning several fields, including medicine and energy generation. Despite the benefits of radiation, precautions must be taken when dealing with radioactive sources as high energy photons can be extremely harmful to the human body [1,2,3,4,5,6].

As a rule of thumb, people should spend the least time in contact with the radiation source as possible and should remain as far away as possible. However, when this is not possible, or when additional measures are needed, shielding provides an effective means to protect workers and patients [7,8,9]. For this purpose, several types of materials are commonly used, depending on the application. To line the walls of X-ray rooms, for instance, concrete is typically used as the absorber, due to its effective attenuation ability of X-rays and its practicality. Although in some cases concrete can be an ideal material, since concrete is prone to cracking and loses its water content after long-term exposure to radiation, other materials are sometimes needed [10,11].

Glasses can be used as radiation shields by incorporating metal oxides into their composition. Heavy metal oxides are typically the most effective, as their high density increases the density of the glass system, which generally correlates with better attenuation features [12,13,14,15,16]. There are three types of metal oxides that can be introduced into a glass composition: a glass former, a glass modifier, and a glass intermediate. Glass formers form the backbone of the glass network, while glass modifiers alter the composition of the glass but do not form part of the backbone of the network.

Phosphate glasses, or glasses containing P_2_O_5_, are currently being used in biomedical applications and as a fast ion conducting materials. Phosphate acts as a network former when introduced to a glass system, forming a polymer-like structure of a regular tetrahedron [PO_4_] ^3−^ groups linked together by covalent bonds in chains and rings. These glasses offer several desirable properties including low optical dispersions, high thermal expansion coefficients and low glass transition temperatures. They are also great attenuators against a wide range of wavelengths, possess a high dielectric constant, and a low phonon energy. Despite these advantages, few studies have been performed investigating the radiation shielding parameters of these glasses [17,18].

Phosphate glasses with no other additives have a low chemical durability and are highly volatile, making pure phosphate glasses no suited for radiation shielding applications. However, other metal oxides can be added to their composition to improve these properties and make them viable for these uses. Metal oxides such as CaO, K_2_O, and Na_2_O act as network modifiers that help stabilize the phosphate glass so its radiation shielding properties can be tested [19,20].

In addition to the phosphate glasses, other kinds of glasses can be used for the radiation protection aims. For instance, heavy metal oxides are among the most common glasses used in this regard. This is because of the high density values of such glass systems, and accordingly the superior attenuation features compared to other glass systems [3,4,5,6,9]. In addition, borate glasses with PbO, Bi_2_O_3_ or BaO are also found to be potentially used for this purposes, especially when incorporating high amounts of the aforementioned oxides [3]. Silicate glasses are also important in the radiation protection field due to the ease of preparation as well as good transmission of light [12].

The mass attenuation coefficient (MAC) is experimentally evaluated for a certain medium to assess the shielding ability of it. The MAC of a medium describes its general shielding capability and factors out its density. The accuracy of the determined MAC values must be precise, as other parameters are calculated from this parameter. Thus, for this purpose, the MAC values are typically compared with the theoretical values of the tested materials and the percent difference is analyzed. Once the MAC results are deemed as reliable, other quantities such as the half value layer (HVL) and mean free path (MFP) are evaluated [21,22,23]. The radiation protection efficiency is widely determined for understanding the ability of the medium to block out photons and thus evaluate the efficiency of the medium as a safe radiation protective material.

In this investigation, the radiation shielding ability for CaO-K_2_O-Na_2_O-P_2_O_5_ glasses were experimentally determined between 0.0595 and 1.41 MeV.

These glasses do not include PbO or Bi_2_O_3_; on the other hand, the current samples are bioactive in nature and include CaO and P_2_O_5_, and these samples are likely to find uses in the medical area where the radiation is employed, thus the radiation shielding characteristics of these glasses are worth reporting. Furthermore, the Ca and K elements’ K-absorption edges occur in the low energy area, which improves the X-ray attenuation properties of these glasses. The experimental MAC was compared with theoretical data obtained from the XCOM to test the accuracy of the experimental values. Additionally, the effect of increasing the P_2_O_5_ in the glass composition, or reducing the Na_2_O content, was evaluated at varying energies.

## 2. Materials and Methods

### 2.1. Methodology for Glasses Preparation

The present glasses have been prepared by the method of the melt quenching. A weighing balance with an accuracy up to four decimal places was used for the present work. To obtain the uniform mixture, the mixing of the P_2_O_5_, Na_2_O, CaCO_3_ and K_2_O oxides is done in an agate mortar. The mixture was then transferred to the alumina crucible. The alumina crucible was placed in a muffle furnace (Purchased from Ambala, Haryana, India.) at 1000 °C to obtain a uniform melt of the mixture. The melted mixture was transferred to a graphite mold for annealing in a muffle furnace temperature 320 °C for 2 h. These samples are used to perform the experimental studies using a narrow beam geometrical setup for measuring the gamma ray shielding parameters. The density of prepared glasses was calculated by Archimedes’ principle [24,25,26]. The samples are coded as:

CaKNaP40: 20 CaO-10 K_2_O-30 Na_2_O-40 P_2_O_5_ (density = 2.334 g/cm^3^)

CaKNaP45: 20 CaO-10 K_2_O-25 Na_2_O-45 P_2_O_5_ (density = 2.286 g/cm^3^)

CaKNaP50 20 CaO-10 K_2_O-20 Na_2_O-50 P_2_O_5_ (density = 2.251 g/cm^3^)

CaKNaP55: 20 CaO-10 K_2_O-15 Na_2_O-55 P_2_O_5_ (density = 2.209 g/cm^3^)

CaKNaP60: 20 CaO-10 K_2_O-10 Na_2_O-60 P_2_O_5_ (density = 2.184 g/cm^3^)

### 2.2. Radiation Attenuation Coefficient Determination

**Figure 1:**
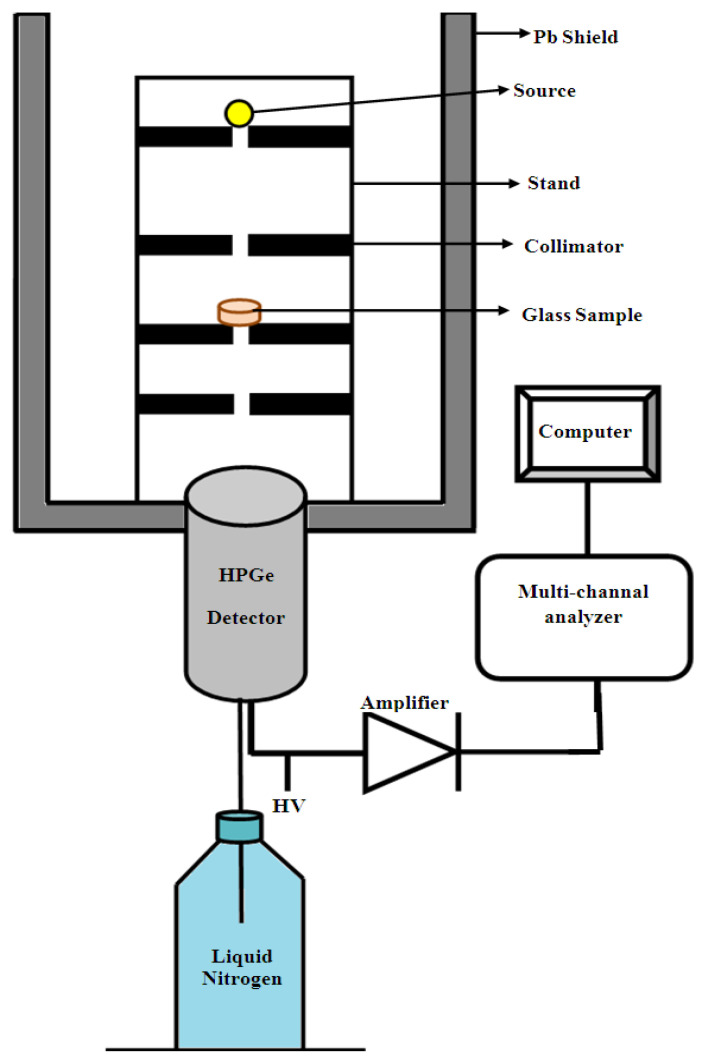
The schematic diagram of the setup used for the determination of the attenuation coefficient.

The main components in this measurement were the detector, radioactive point source, lead collimator and the glass samples or an absorber needed for measuring. The schematic diagram of these components is illustrated in Figure 1. The High Pure Germanium (HPGe) Detector of model: GC1520 (Manufacturer of radiation detection and analysis instrumentation, Meriden, USA) was used. The relative efficiency of the detector was 15% and the resolution (FWHM) at 1.33 MeV was 2 keV. Three point sources were used in the measurement to cover a wide range of energies. The Am-241 point source is a very important source that emits a line in low energy (59.54 keV), and the initial activity of this source was 259 kBq. Cs-137 point source emits two lines (32, 661.6 keV) but the line due to X-ray emission (32 keV) was totally absorbed and therefore the most probable line and higher emission probability is (661.6 keV), the initial activity of this source is 385 kBq. Eu-152 is a multi-line source and covered more energies from low to high energy. The lines were chosen according to the higher emission probability (121.78, 244.69, 344.28, 964.13 and 1408.1 keV), the initial activity of this source is 290 kBq. The reference date of all three point sources was 1 June 2009 [27,28].

The narrow beam method was used in measurements by the lead collimator. The detector first was calibrated and the background was measured using Genei 2000 software (V3.3, Mirion Technologies (Canberra), Inc., Canberra, Australia) [29]. The measurement occurred within and outside the glass sample with different sources to obtain the net peak area or the count rate of the line which represent the intensity of this line. So, the intensity of the line outside the absorber (*I*_0_) and within the absorber (*I*) can be calculated. By knowing the thickness of the glass absorber (*x*), the linear attenuation coefficient (*LAC*) easily estimated via the relation [30].

(1) LAC=−ln(II0)x

The MAC can be calculated experimentally by dividing the *LAC* on the density of an absorber (*ρ*). The MAC examined theoretically by the XCOM program for all present glass samples using the chemical composition of each sample. Other related parameters were investigated such as HVL, TVL, and MFP, as well as RPE. The HVL and TVL are the thickness layers of an absorber needed to reduce the count rate of a line a half and a tenth of its original value, respectively, and are given by the following equations [31].

(2) $$\mathrm{HVL}=\frac{Ln2}{LAC}$$

(3) $$\mathrm{TVL}=\frac{Ln10\;}{LAC}$$

The mean-free path can be estimated by Equation (4) [32].

(4) $$\mathrm{MFP}=\frac{1}{LAC}$$

The shielding efficiency of an absorber sample can be investigated using a parameter called the radiation protection efficiency (RPE) and given by the next equation [33].

(5) $$\mathrm{RPE}=(1-\frac{I}{I_{0}})\times 100$$

## 3. Results and Discussion

**Figure 2:**
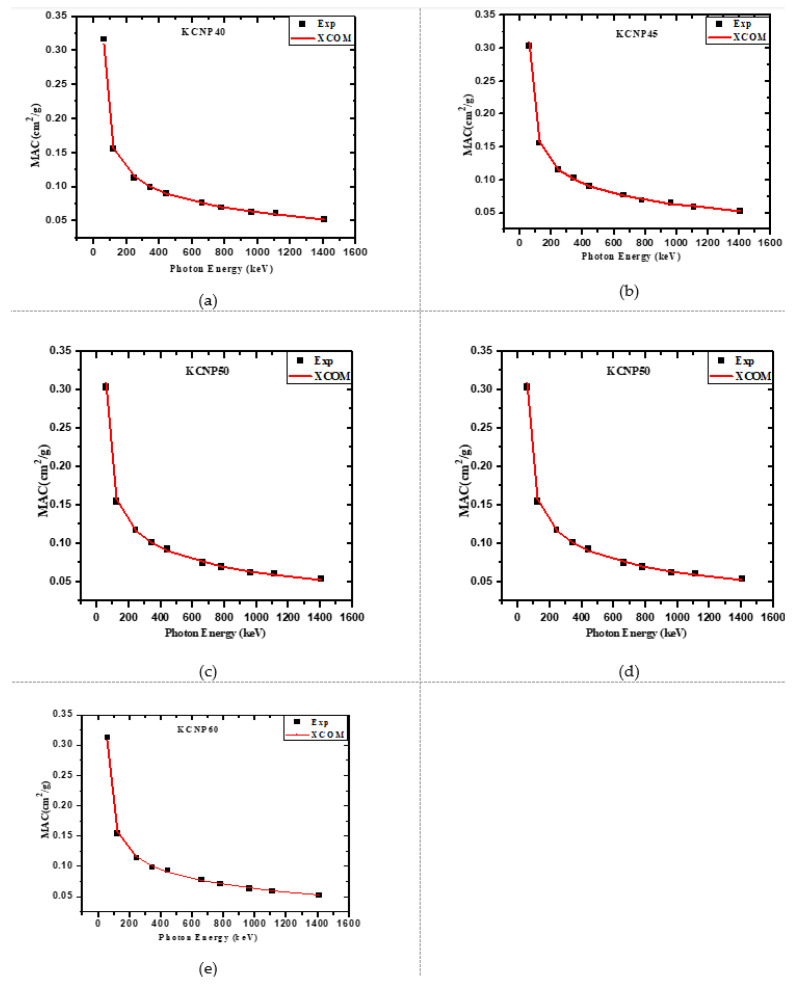
The comparison between the MAC obtained by experimental method and calculated by XCOM (all these are one figure). (**a**) CKNP40; (**b**) CKNP45; (**c**) CKNP50; (**d**) CKNP55; (**e**) CKNP60.

**Figure 3:**
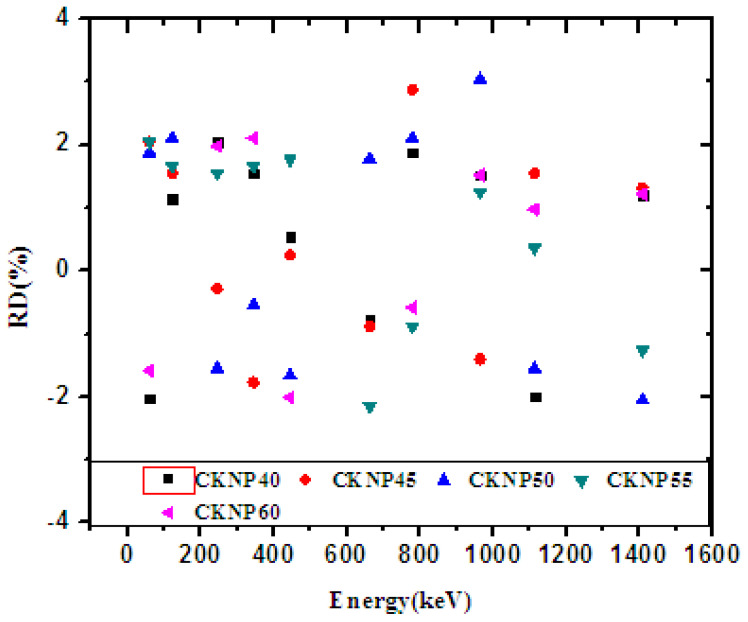
The relative difference between the mass attenuation coefficient obtained by experimental method and calculated by XCOM.

In Figure 2, the experimental mass attenuation coefficient (MAC) was compared with the theoretical XCOM data against increasing energy for the five fabricated glasses. Additionally, the values for both methods are given in Table 1. The aim of this comparison is to validate the experimental method, an important step, as the experimental MAC results will be used to determine the other parameters. In the four subfigures, the experimental results are represented by a black square and the XCOM results by the red line. For the CKNP40, CKNP45, CKNP50, CKNP55, and CKNP60 glasses, the experimental data strongly agreed with the XCOM results. The results in Table 1 imply that the experimental setup is highly precise and accurate and is an effective way to determine the MAC of these glasses. To illustrate the percent difference between the results, the relative difference between the MAC determined by the two methods was graphed in Figure 3. The figure suggests that for all glasses analyzed, the percent difference between the values is within 2%. This figure reaffirms the validity of the experimental method used to obtain the MAC values. The high MAC of these glasses in the low energy region is due to the presence of a K-absorption edge for some elements in the composites such as Ca and K at low energies. In addition, at low energies, the photoelectric effect is the dominant one.

**Figure 4:**
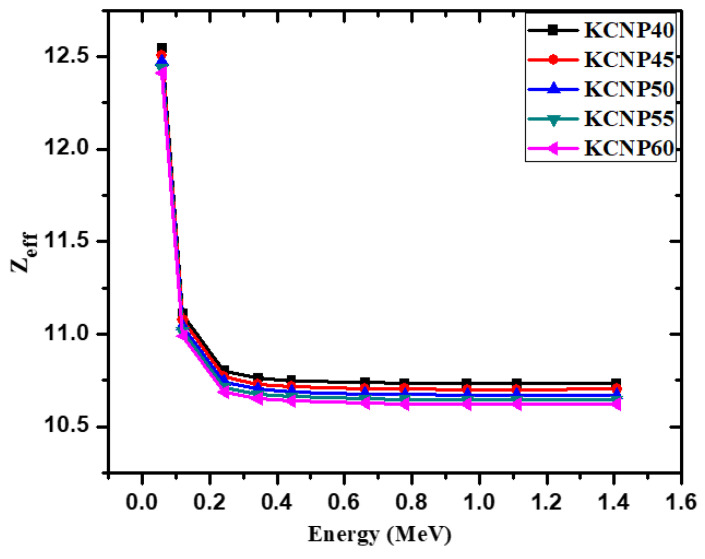
The effective atomic number for the fabricated glasses.

Figure 4 demonstrates the effective atomic number (Z_eff_) of the chosen glasses. The Z_eff_ values start out at their maximum (12.41–12.55) at 0.0595 MeV and decrease to 10.69–10.80 at 0.245 MeV. At low energies, the values are at their highest thanks to the photoelectric effect. As energy further increases, the Z_eff_ values remain almost constant between 0.344 and 1.41 MeV. This relatively constant trend can be explained by the elements of the fabricated glasses (Ca, K, Na, P and O), which have close atomic numbers. This result is consistent with recent studies evaluating the Z_eff_ of ceramic containing Mg and Si, which they also found to be constant at these energies [34]. At all energies, the Z_eff_ values are between 10.66 and 12.55, which makes sense since the lowest atomic number within the composition is 8 for O and the highest is 20 for Ca.

**Figure 5:**
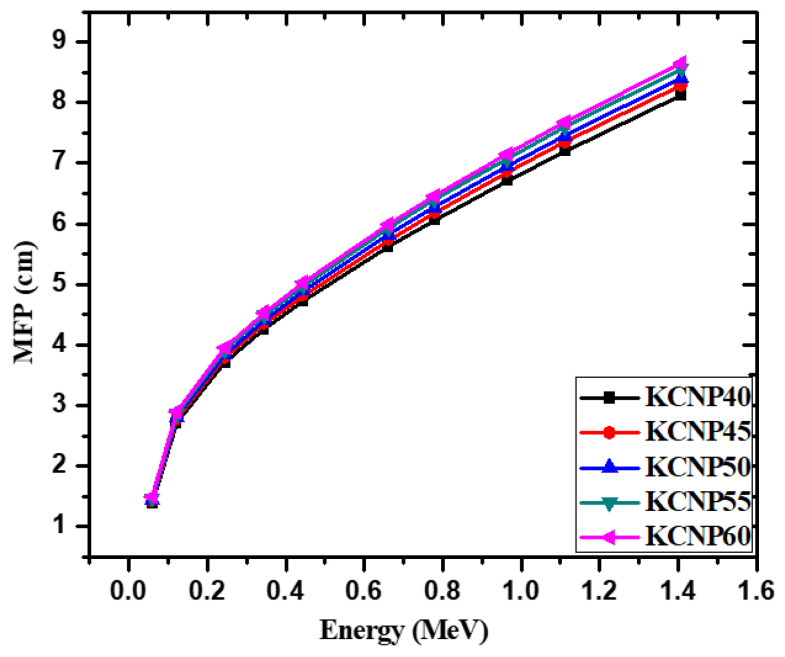
Mean free path for the fabricated glasses.

The mean free path (MFP) of the fabricated glasses is plotted in Figure 5. The lowest MFP value occurs at the lowest tested energy, 0.0595 MeV, and lies within the range of 1.382–1.486 cm, while the greatest MFP can be found at 1.41 MeV, within the range of 8.121–8.656 cm. This upward trend occurs because higher energy radiation can penetrate the incident material easily. At higher energies, the dominance of the photoelectric effect decreases compared to the Compton interaction [35]. This alteration causes a decrease in photon attenuation since the Compton interaction is weakly dependent on the energy (E) as well as the atomic number, and only occurs between the incoming photons and an outer shell electron of an atom within the glasses. In addition, at all energies, the KCNP40 sample has the lowest MFP, while the KCNP60 sample has the greatest. For example, at 0.245 MeV, KCNP40 has an MFP equal to 3.710 cm and KCNP60 has an MFP equal to 3.956 cm. Meanwhile, at 1.11 MeV, the MFP’s of KCNP40 and KCNP60 are equal to 7.202 and 7.676 cm, respectively. These results are directly related to the density of the glasses, as more interactions will occur between photons and the atoms of a denser material, increasing attenuation. In other words, increasing the density of a material causes an increase in the chances of interaction between the incoming radiation and the shield.

**Figure 6:**
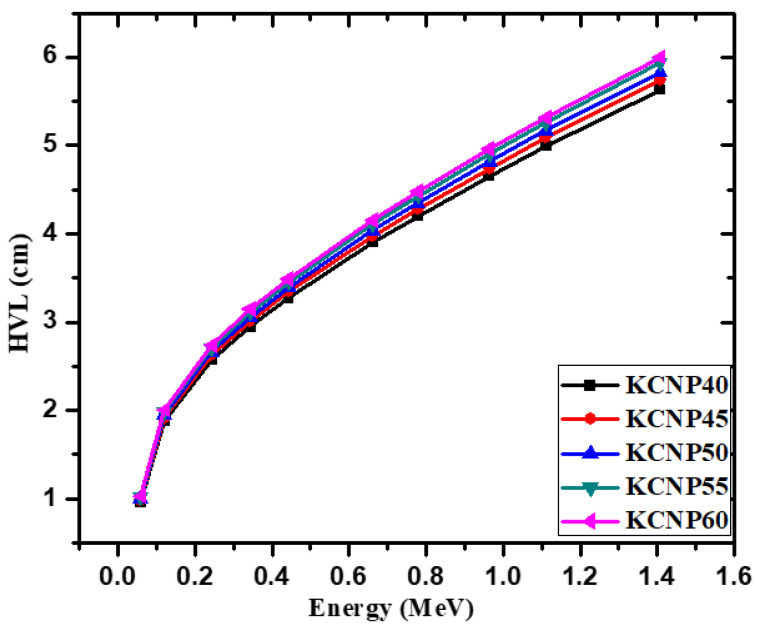
Half value layer for the fabricated glasses.

The half value layer (HVL) is a common factor used in the radiation shielding studies [36]. A lower HVL represents a more space-efficient, and thus better, radiation shield [37]. In Figure 6, we graphed the HVL for the KCNP-X glasses. For all the selected energies, the HVL values follow the order of KCNP40 < KCNP45 < KCNP50 < KCNP55 < KCNP60. In other words, the KCNP40 glass has the lowest HVL, and the best shielding ability, while the KCNP60 sample has the highest HVL, and the least desirable shielding ability. Additionally, the influence of the HVL against increasing energy was analyzed. It was observed that the HVL increases with raising the energy. The HVL of the KCNP50 sample increased from 0.996 to 2.663, 3.392, 4.351, and 5.169 cm for energies of 0.0595, 0.245, 0.444, 0.779, and 1.11 MeV, respectively. This increasing trend occurs because photons with higher energies collide less often with atoms in the material and tend to pass through the glasses more often, which leads to an increase in HVL. Thus, the glasses are more space-efficient at lower energies but are less space-efficient at higher energies.

**Figure 7:**
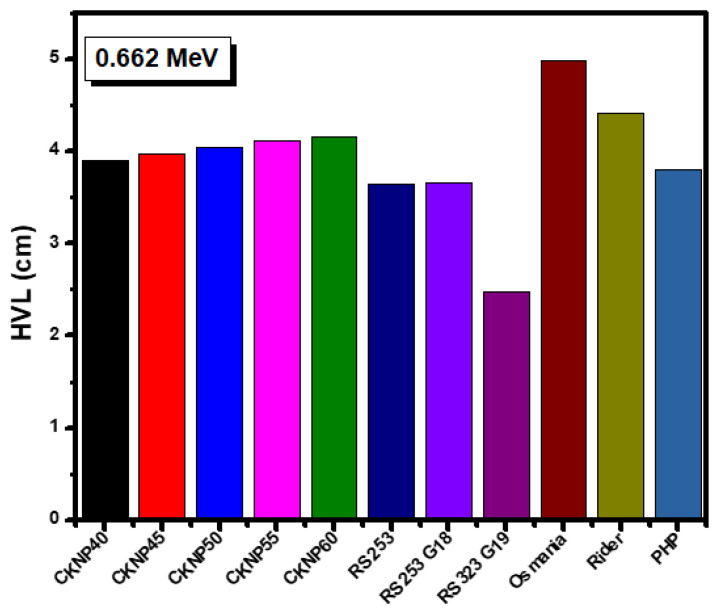
Comparison between the HVL for the tested glasses with other shielding glasses.

In Figure 7, we compared the HVL for the KCNP-X glasses with other glasses at 0.662 MeV. The glasses used for the comparison is reported in [38,39]. Apparently, all the KCNP-X glasses have lower HVLs than rider and Osmani glasses, while PHP and RS 253 have almost the same HVL as KCNP40 and RS 323 G19 possesses a lower HVL than all the KCNP-X glasses.

**Figure 8:**
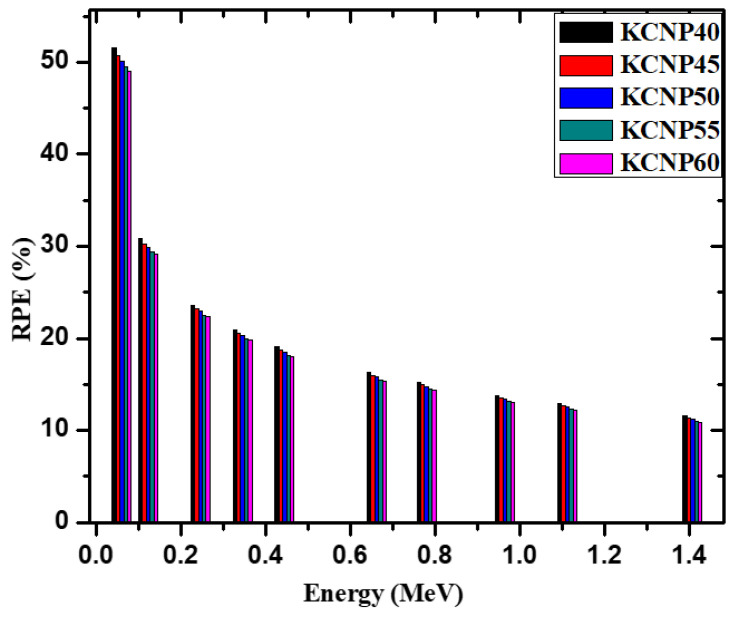
The radiation protection efficiency for the fabricated glasses.

The radiation protection efficiency (RPE) of the KCNPX samples is graphed against increasing photon energy in Figure 8. For all tested energies, RPE decreases with increasing energy [40]. For the KCNP45 sample, for example, its RPE decreases from 51% at 0.0585 MeV to 23% at 0.245 MeV, 19% for E = 0.444 MeV, 15% for E = 0.779 MeV, and 12% for E = 1.41 MeV. For the KCNP55 sample, meanwhile, its RPE is equal to 49%, 23%, 18%, 14%, and 11% for the previous energies. This decreasing behavior occurs since higher energy photons penetrate via the samples easily, decreasing the amount of photons attenuation by the shields, and decreasing RPE. This result also indicates that the KCNPX specimens are more efficacious against lower energy photons. When the RPE values are evaluated at a single energy, the KCNP40 sample has the greatest RPE, while the KCNP60 sample has the least. Finally, since the KCNP40 sample has the greatest Na_2_O content, this glass has the best possibility for radiation shielding utilizations.

## 4. Conclusions

The radiation shielding ability for CaO-K_2_O-Na_2_O-P_2_O_5_ glasses was experimentally examined between 0.0595 and 1.41 MeV. The experimental data strongly agreed with the XCOM results. The Z_eff_ values start out at their maximum (12.41–12.55) at 0.0595 MeV and decrease to 10.69–10.80 at 0.245 MeV. The lowest MFP value occurs at the lowest tested energy, 0.0595 MeV, and lies within the range of 1.382–1.486 cm. The KCNP40 sample has the lowest MFP, and the KCNP60 sample has the greatest MFP. The values follow the order of KCNP40 < KCNP45 < KCNP50 < KCNP55 < KCNP60. The HVL of the KCNP50 sample increased from 0.996 to 5.169 cm between 0.0595 and 1.11 MeV. The RPE demonstrated that decreasing the P_2_O_5_ content in the glasses improves the radiation shielding ability of the samples. Thus, KCNP40 has the most potential for radiation protection applications.

## Figures and Tables

**Table 1:** The measured mass attenuation coefficient and XCOM results for the fabricated glasses.

Energy (keV)	CKNP40		CKNP45		CKNP50		CKNP55		CKNP60	
	Experimental	XCOM	Experimental	XCOM	Experimental	XCOM	Experimental	XCOM	Experimental	XCOM
59.54	0.317 ± 0.030	0.310	0.303 ± 0.022	0.310	0.303 ± 0.038	0.309	0.302 ± 0.041	0.309	0.313 ± 0.022	0.308
121.80	0.156 ± 0.011	0.158	0.155 ± 0.011	0.158	0.155 ± 0.026	0.158	0.155 ± 0.033	0.158	0.052 ± 0.030	0.158
244.70	0.113 ± 0.009	0.116	0.116 ± 0.025	0.116	0.117 ± 0.014	0.116	0.114 ± 0.038	0.116	0.113 ± 0.028	0.116
344.30	0.099 ± 0.021	0.101	0.103 ± 0.040	0.101	0.101 ± 0.025	0.101	0.099 ± 0.022	0.101	0.099 ± 0.021	0.101
444.00	0.090 ± 0.035	0.091	0.091 ± 0.009	0.091	0.092 ± 0.014	0.091	0.089 ± 0.030	0.091	0.093 ± 0.019	0.091
661.70	0.077 ± 0.007	0.076	0.077 ± 0.026	0.076	0.075 ± 0.022	0.076	0.078 ± 0.025	0.076	3.819 ± 0.017	0.076
778.90	0.069 ± 0.008	0.071	0.069 ± 0.031	0.071	0.069 ± 0.027	0.071	0.071 ± 0.018	0.071	0.071 ± 0.028	0.071
964.10	0.063 ± 0.025	0.064	0.065 ± 0.030	0.064	0.062 ± 0.013	0.064	0.063 ± 0.012	0.064	0.063 ± 0.012	0.064
1112.00	0.061 ± 0.009	0.059	0.059 ± 0.024	0.060	0.061 ± 0.009	0.060	0.059 ± 0.014	0.060	0.059 ± 0.010	0.060
1408.00	0.052 ± 0.011	0.053	0.052 ± 0.009	0.053	0.054 ± 0.010	0.053	0.302 ± 0.041	0.309	0.313 ± 0.022	0.308

## Data Availability

The data presented in this study are available on request from the corresponding author.
